# Dosimetric Comparison of Various Spot Placement Techniques in Proton Pencil Beam Scanning

**DOI:** 10.14338/IJPT-21-00022.1

**Published:** 2022-01-31

**Authors:** Mahboob ur Rehman, Omar A. Zeidan, Twyla Willoughby, Sanford L. Meeks, Patrick Kelly, Kevin Erhart

**Affiliations:** 1 University of Central Florida (UCF), Orlando, FL, USA; 2 Orlando Health Cancer Institute, Orlando, FL, USA; 3.decimal LLC, Sanford, FL, USA

**Keywords:** proton therapy, spot placement in proton pencil beam scanning

## Abstract

**Purpose:**

To present quantitative dosimetric evaluations of five proton pencil beam spot placement techniques.

**Materials and Methods:**

The spot placement techniques that were investigated include two grid-based (rectilinear grid and hexagonal grid, both commonly available in commercial planning systems) and three boundary-contoured (concentric contours, hybrid, and optimized) techniques. Treatment plans were created for two different target volumes, one spherical and one conical. An optimal set of planning parameters was defined for all treatment plans and the impact of spot placement techniques on the plan quality was evaluated in terms of lateral/distal dose falloff, normal tissue sparing, conformity and homogeneity of dose distributions, as well as total number of spots used.

**Results:**

The results of this work highlight that for grid-based spot placement techniques, the dose conformity is dependent on target cross-sectional shape perpendicular to beam direction, which changes for each energy layer. This variable conformity problem is mitigated by using boundary contoured spot placement techniques. However, in the case of concentric contours, the conformity is improved but at the cost of decreased homogeneity inside the target. Hybrid and optimized spot placement techniques, which use contoured spots at the boundary and gridlike interior spot patterns, provide more uniform dose distributions inside the target volume while maintaining the improved dose conformity. The optimized spot placement technique improved target coverage, homogeneity of dose, and minimal number of spots. The dependence of these results on spot size is also presented for both target shapes.

**Conclusion:**

This work illustrates that boundary-contoured spot placement techniques offer marked improvement in dosimetry metrics when compared to commercially available grid-based techniques for a range of proton scanned beam spot sizes.

## Introduction

Intensity-modulated proton therapy (IMPT) using a pencil beam scanning technique is an advanced method of radiation delivery in which small proton beamlets (pencil beams) are magnetically scanned to the appropriate positions in the patient such that an optimal dose can be delivered by controlling the strength and location of the individual beamlets. Relatively less entrance dose, uniform coverage of the tumor, and rapid dose falloff in both lateral and distal directions are the main dosimetric characteristics that are advantageous over other treatment modalities [1–3]. These properties allow proton beams to treat a wide variety of complex-shaped tumors at different anatomic locations while providing superior sparing to nearby organs at risk. There are several beam delivery parameters that impact IMPT plan quality, including layer spacing, spot size, and spot locations within each layer [4–10]. These parameters can vary with the energy of the pencil beam, its depth in tissue, and each vendor's specific delivery system [11, 12]. Spot spacing is defined within the treatment planning systems, often as a function of beamlet width that is defined in terms of full width at half maximum (FWHM) in air at isocenter. Layer spacing is also defined within the treatment planning system but limited energies may be available, based on proton machine type. Layer spacing typically affects dose ripples along the depth axis and is often set to match the distal and proximal 80% doses of the pristine Bragg peak width. Spot sizes on the other hand are a property of the proton delivery equipment and cannot be altered by the treatment planner. Generally, smaller spot sizes are more favorable, producing better and more conformal treatment plans [5, 7, 9, 10, 13]. In a similar manner, spot placement within an energy layer is also an important treatment planning parameter that can impact plan quality in terms of lateral dose falloff, dose conformity, and reduced integral dose to organs at risk [4, 5, 8, 14]. However, little attention has been given to single out the dosimetric effects of spot placement algorithms within each layer.

In commercially available treatment planning systems, spots are distributed on a regular (rectilinear or hexagonal) starting grid. The grid can be a 2D or 3D grid depending on the vendor. Eclipse (Varian Medical Systems, Palo Alto, California) uses a 3D grid with adjacent layers of spots aligned and a fixed spot spacing for all energy layers [4, 15], although options to adjust spacings per layer are available. Pinnacle (Philips Medical Systems, Bothell, WA, USA) uses a 2D grid for each energy layer with adjacent layers of spots offset by half the spot spacing. RayStation (RaySearch Laboratories, Stockholm, Sweden) and XiO (Elekta Solutions AB, Stockholm, Sweden) can use either 3D or 2D grids depending on the use of fixed or variable spot spacing [15–19]. The grids defined for spot placement are orthogonal to the direction of the incident proton beam. As the spots are distributed on fixed sized grids, spots slightly outside of the selected target volume must often be included to ensure full coverage all the way to the target edges [4, 20]. Failure to select these external spots can result in dose undercoverage of the target. The same is true when using a hexagonal grid for spot placements [13]. This led to the idea of proposing alternative spot placement techniques that selectively place spots directly on (or at a fixed distance relative to) the geometric boundary of the target and then fill in the spots internally as required depending on the shape of the target. Note this must be done uniquely for each layer along the beam direction, as the target shape can vary significantly with radiologic depth. Several such techniques have been previously reported as concentric contours, hybrid, and optimized spot placement techniques and have shown improvement in the treatment plan quality in terms of the dose falloff, reduced number of spots, and more efficient and faster delivery [2, 6, 8, 13]. In all 3 of these approaches, the spot placement process begins with an outer set of spots that closely follow the perimeter of the target projection (note that only the portion of the target within the high-dose region of the Bragg peak is included in this projection for each energy layer, that is, the target is sliced into regions of constant radiologic depth to determine the appropriate shape onto which the spots should be distributed). After placing this outer band of spots, the techniques differ, with (1) the concentric contours continuing in the same fashion by distributing spots following the shape of the outer perimeter but converging inward with each subsequent band of spots until reaching the center; (2) the hybrid approach filling the interior with a rectangular or hexagonal grid up to the point of overlap with the outer band of perimeter placed spots; and (3) the optimized approach first filling the interior with a hexagonal grid and then using an iterative approach to optimally redistribute the interior spots to achieve uniform interspot distances throughout the entire target projection [8]. **Figure 2** shows spot distributions that result from each of these approaches.

This work does not introduce any new spot placement approaches, but instead is a follow-up to the earlier study by the same authors [8] in which this novel optimized spot placement technique was introduced. Prior work compared the new approach to existing spot placement algorithms geometrically and in 2D space for a single energy layer (perpendicular to beam direction). Those results demonstrated that the concentric-contours approach suffers from significant spot nonuniformity in the interior regions, that the hybrid approach suffers from significant nonuniformity at the interface between the interior grid and the boundary contoured spot band, and that the new optimized approach was able to mitigate both of these issues and provide nearly constant interspot spacing throughout the entire target shape. However, prior work did not include any studies to determine if these improvements would extend to actual dosimetric benefit to patients. Herein we address this deficiency and include realistic 3D dose calculations and analyze clinically relevant dosimetric metrics for each spot placement technique with multiple energy layers for 2 different 3D target shapes. The results presented herein should provide the reader with a better understanding of the impact of each spot placement technique on the dose distribution within and in the immediate vicinity of actual 3D target volumes. These effects are quantified in terms of maximum dose inside the target, dose falloff in both lateral and distal directions (penumbra), dose conformity in terms of conformity index (CI), dose homogeneity in terms of homogeneity index (HI), and number of spots used to achieve these optimal delivery metrics.

## Materials and Methods

The Astroid treatment planning system (.decimal LLC, Sanford, Florida) was used to develop treatment plans in a cubical homogeneous water phantom for 2 target cases. In the first case, a spherical target volume of 50-mm diameter centered at a depth of 75 mm from the surface of the phantom in the beam direction was used. The second case has a conical target volume with a base diameter of 62 mm and height of 60 mm. The base of the cone is placed at a depth of 90 mm from the surface of the phantom in the beam direction (**Figure 2a**). The spherical target volume serves as a useful tool to study the dose falloff in terms of lateral and distal penumbra as well as dose conformity with minimal complexity. The conical target volume is used to highlight certain dosimetric aspects of the treatment plans in the dose falloff region when the boundary of the target is continuously changing along the energy layers. Two distinct spot sizes were used to provide a realistic analysis, based on the known spot sizes of common commercially available proton delivery systems. The larger spot size has an in-air sigma of 5.8 mm defined at isocenter for 145-MeV energy. This spot size is in the mid to high range of typical pencil beam scanning spot sizes for active proton therapy centers at moderate energies (∼145 MeV). A smaller spot size (in-air sigma of 4.3 mm at isocenter for 145 MeV) is also used to see the impact of the spot placement technique in conjunction with reduced spot size.

Since the final dose distributions in the target volumes have a strong dependency on the dose optimization parameters, we defined and implemented a set of common constraints in combination with dose-volume-histogram (DVH) based objectives to provide a fair comparison between the spot placement techniques using single field uniform dose optimization in ASTROID treatment planning system. Lateral and distal margins for the spot placements were set at 13 mm and 10 mm, respectively, for all spot placement algorithms, while the spot spacing was set to be 85% the spot sigma in air at isocenter. A fine dose grid of 2 mm × 2 mm × 2 mm was created over the area of interest to ensure the dose gradients and penumbra region dose are accurately captured. The geometric center of both target volumes was selected as isocenter for the beam and an air gap of 50 mm was used.

To ensure a fair comparison, each plan was constrained to achieve full (100%) coverage of the tumor volume by the prescription dose and a global maximum dose of 112% was allowed. It should be noted that setting a reasonable maximum dose is very important for this study, as it provides each spot placement technique with the same opportunity to take advantage of the edge-enhancement effect previously described by Pedroni et al [2]. Astroid's advanced Multi-Criteria Optimization (MCO) tool [16, 18, 21] was configured with several competing objectives designed to allow for efficient exploration of the trade-offs between target coverage, maximum dose, and dose conformity. The MCO controls were used to drive each plan to a dose field that achieved the best possible combination of the metrics identified below while not violating the constraints described above. The following metrics were calculated to evaluate the overall treatment plan quality:

- D_max_: Maximum dose inside the patient

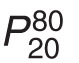
: Dose falloff from 80% to 20% in both lateral and distal directions (Penumbra)
- Conformity index (CI): Ratio between the volumes covered by the reference isodose and the target volume [22]
- Homogeneity index (HI): Ratio between the dose reached in 95% of the target volume (D_95_) and the dose reached in 5% of the target volume (D_5_)
- Integral dose (ID): Mean dose multiplied by the volume of the structure
- Total spot count (spots with monitoring units below 0.01% of the total meterset were ignored)

## Results

**Figure 1.**
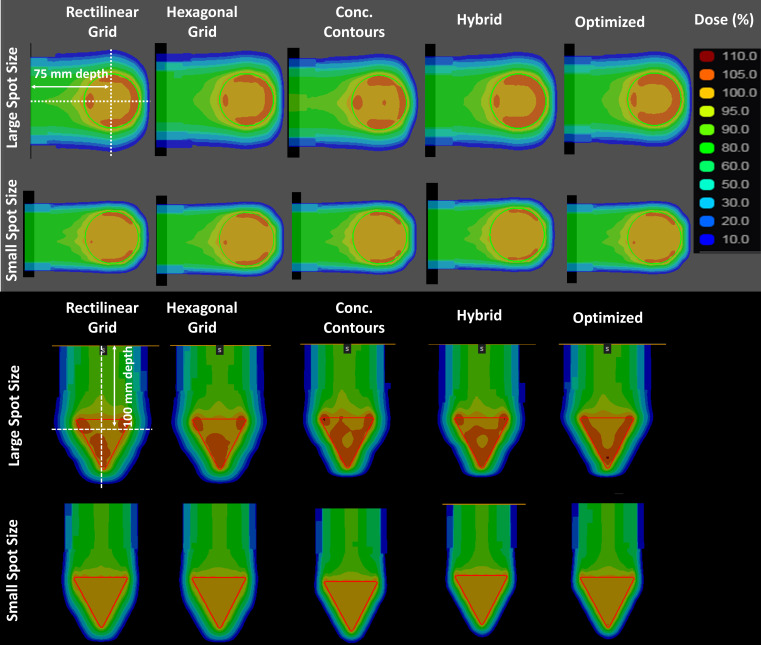
Dose distributions from all five spot placement techniques for spherical target volume using large spot size (row 1), spherical target volume using small spot size (row 2), conical target volume using large spot size (row 3), and conical target volume using small spot size (row 4). The beam is incident from the left (rows 1 and 2) and from the top (rows 3 and 4).

**Figure 2.**
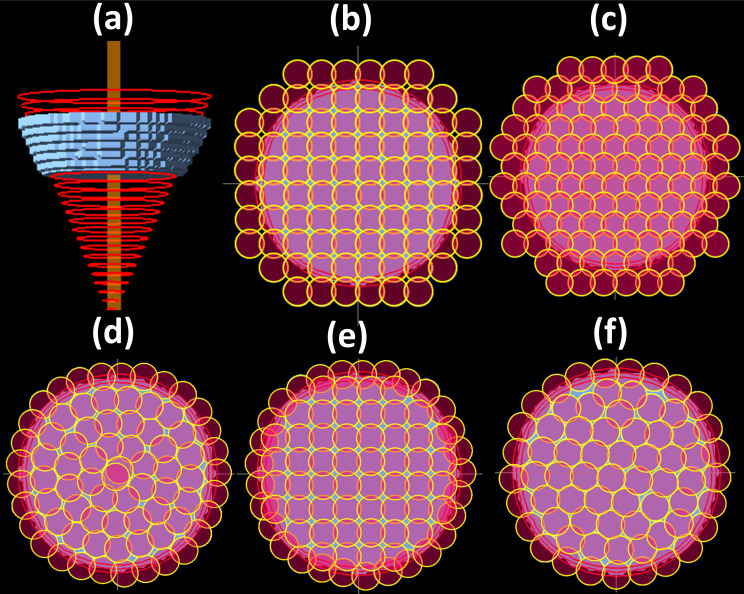
Spatial distribution of spots using all available spot placement techniques for the conical target volume at an energy layer of 121.77 MeV shown as a blue stripe (a), using rectilinear grid (b), hexagonal grid (c), concentric contours (d), hybrid (e), and optimized (f).

The computed dose distributions for both the spherical and conical target volumes are shown in **Figure 1** for all spot placement techniques using both spot sizes. **Figure 1** shows a subtle visual improvement in dose conformity when using the boundary-contoured spot placement techniques compared to the conventional grid-based spot placement techniques. This is particularly evident in row 3 of **Figure 1** for the conical shape target. The increased level of conformity is also reflected in the CI values in **Tables 1** and **2** for both target shapes. Using a smaller spot size, however, the dose conformity is minimally improved in case of boundary-contoured spot placement techniques, but the dose homogeneity level, as reflected by the HI in **Tables 1** and **2**, is similar for all spot placement techniques. The spatial distributions of the larger spots for the 121.77-MeV energy layer are shown in **Figure 2** for all spot placement techniques and the portion of the target volume used for boundary contouring for this energy layer is shown in **Figure 2a**. In the spherical target volume case, 9 energy layers were used and for conical target volume, 11 energy layers were used. The total numbers of spots across all layers are provided in **Tables 1** and **2** for each target, using large and small spot size. The spots are represented by semitransparent red circles to highlight the areas of spot overlap. **Figure 2** shows improved levels of spatial conformity with contour-based techniques over boundary-based techniques. This is evident from the tighter packing patterns with less voids and overflow of spots outside the target as seen in **Figure 2d** through **2f** compared to **Figure 2b** and **2c**. **Figure 2f** also shows how the optimized spot placement technique achieves high levels of both conformity (minimal spot coverage outside target) and uniformity (consistent spot overlap throughout the target and no voids) in the spatial distribution of spots. It can also be seen that extra spots are required to fully cover the target when using grid-based spot placement techniques, as some spots fall just enough inside the target edge such that low dose at the target edges will be seen if additional spots are not used.

**Table 1.** Evaluation metrics for the spherical target volume using large and small spot sizes for different spot placement techniques.

**Spot placement technique**	**D_max_ (Gy)**	**Lateral dose falloff (mm)**	**Distal dose falloff (mm)**	**Conformity index**	**Homogeneity index**	**Integral dose (Gy-L)**	**Spots count**
Large spot size (5.8 mm)							
Rectilinear grid	108.44	12.14	5.00	1.6	1.08	14.97	636
Hexagonal grid	107.8	12.12	5.04	1.6	1.08	15.24	741
Concentric contours	109.9	10.98	5.10	1.58	1.1	14.16	394
Hybrid	109.82	11.00	5.07	1.56	1.1	14.16	434
Optimized	109.10	10.93	5.10	1.57	1.09	14.16	360
Small spot size (4.3 mm)							
Rectilinear grid	109.54	5.9	5.36	1.45	1.10	10.34	2841
Hexagonal grid	109.77	5.82	4.91	1.44	1.10	10.34	3288
Concentric contours	108.22	6.08	4.92	1.45	1.08	10.62	1473
Hybrid	108.31	5.89	5.19	1.44	1.08	10.34	1558
Optimized	108.79	5.96	4.92	1.44	1.09	10.34	1303

**Table 2.** Evaluation metrics for the conical target volume using large and small spot sizes for different spot placement techniques.

**Spot placement technique**	**D_max_ (Gy)**	**Lateral dose falloff (mm)**	**Distal dose falloff (mm)**	**CI^a^**	**Homogeneity index**	**Integral dose (Gy-L)**	**Spots count**
Large spot size (5.8 mm)							
Rectilinear grid	108.85	13.30	4.90	2.47	1.09	70.51	580
Hexagonal grid	109.83	13.02	4.95	2.15	1.10	65.88	602
Concentric contours	110.60	10.90	2.71	2.11	1.11	54.99	394
Hybrid	110.56	11.05	2.70	2.01	1.11	54.72	420
Optimized	110.12	11.80	2.67	1.99	1.10	57.71	358
Small spot size (4.3 mm)							
Rectilinear grid	105.06	8.75	9.61	1.94	1.05	51.72	1799
Hexagonal grid	104.06	9.46	9.52	1.95	1.04	52.27	2136
Concentric contours	106.74	7.99	7.25	1.74	1.07	42.74	1393
Hybrid	107.12	7.79	8.37	1.74	1.07	42.47	1459
Optimized	108.41	7.88	7.13	1.74	1.08	41.92	1223

**Abbreviation:** CI, conformity index.

a The value of CI is towards higher level because of the use of single field for a larger volume.

**Figure 3.**
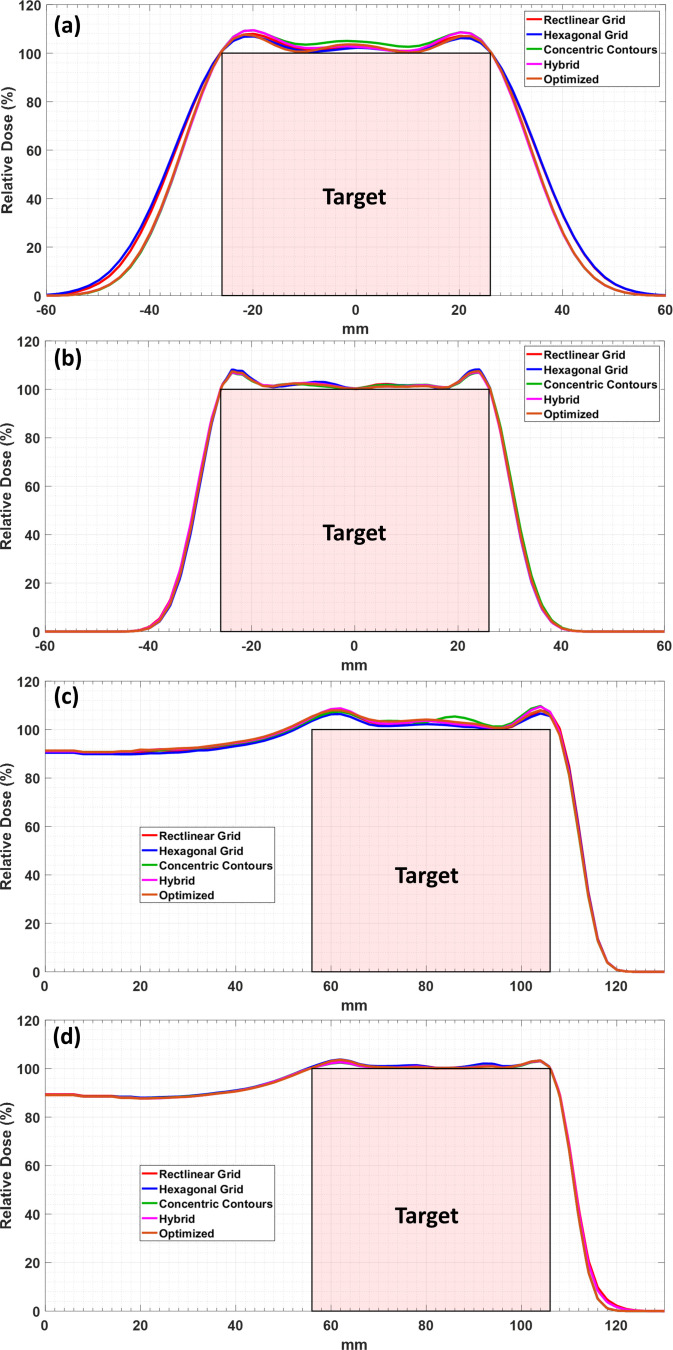
Dose profiles for the spherical target volume using all spot placement techniques. Lateral and central axis dose profiles using large spot size (a, b), and central axis depth dose profiles using small spot size (c, d).

**Figure 4.**
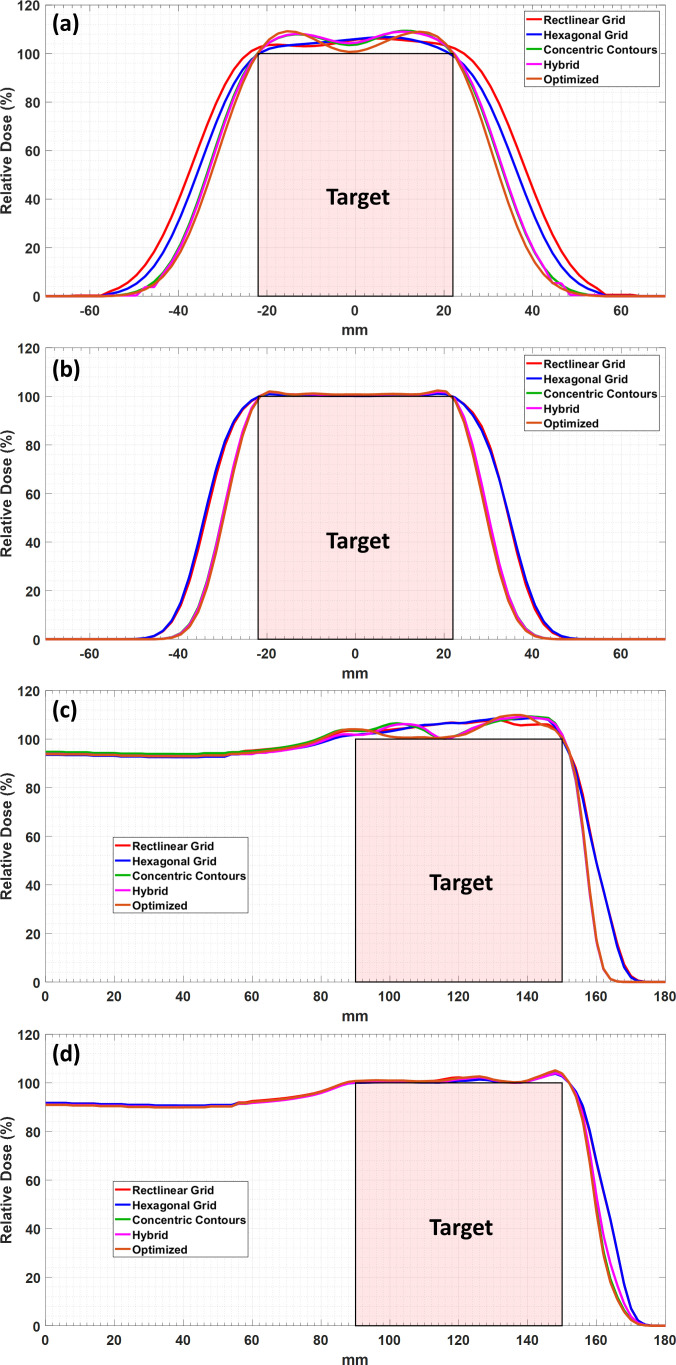
Dose profiles for the conical target volume using all spot placement techniques. Lateral and central axis dose profiles using large spot size (a, b), and central axis depth dose profiles using small spot size (c, d).

Lateral dose profiles are used to compute the penumbra for each case with representative profiles shown in **Figures 3** and **4** (note that the tumor extents are shown as a red box to allow visual assessment of the lateral and distal conformity of each profile in these figures). These profiles were taken at depths of 75 mm and 100 mm from the surface of the water phantom for the spherical and conical target volumes, respectively. These positions are shown in **Figure 1** by white dotted lines. The differences between contour-based and grid-based techniques are more pronounced in the lateral profiles shown in **Figure 3** for the larger spot size. The differences are minimal or indistinguishable for the smaller spot size for both the lateral and distal profiles. **Figure 4** shows the same trend for the cone-based target, with the contour-based techniques consistently showing improved penumbras and conformity over the grid-based techniques. However, it seems that the difference is more pronounced with the cone-based plan than the spherical plan. It is worth noting that the optimized technique shows the most conformity and the best penumbra (lateral and distal) of all other techniques.

A detailed dosimetric comparison for both target volumes was performed by computing a set of metrics from the treatment plan dose distributions in the Astroid treatment planning system. **Tables 1** and **2** list the metrics computed for the spherical and conical target volumes, respectively, using all available spot placement techniques for both spot sizes. The lateral and distal falloff is either improved for boundary-contoured spot placement techniques or remains consistent with the grid-based spot placement techniques. The concentric contours show a marked decrease in dose homogeneity inside the spherical target volume, while hybrid and optimized techniques show only small decreases in homogeneity. There is a clear reduction in the total number of spots required for treatment of the whole target volume for all boundary-contoured techniques (**Tables 1** and **2**). Clear improvements are seen for the larger spot size cases; however, the impact on plan quality is much less pronounced for the smaller spot size cases. For the conical target volume, lateral dose falloff, CI, and total dose to the normal tissue are improved for the larger spot size with the boundary-contoured techniques, but at the cost of increased maximum dose. It should be noted that attempts were made to improve the falloff for the grid-based techniques, but the placement of spots was not conducive to taking advantage of edge-enhancement effects, therefore such attempts did not yield improvements in other metrics as are shown in the results for the boundary-contoured techniques.

## Discussion

In this work, the concept of using boundary-contoured spot placement techniques has been explored as an alternative to traditional grid-based techniques to improve IMPT plan quality. The dosimetric study performed herein has highlighted that superior levels of dose conformity in both lateral and distal directions can be achieved by using boundary-contoured spot placement techniques as compared to grid-based distributions under similar conditions. For the case of a concentric-contour spot placement technique it was shown that these benefits were achieved at the cost of increased maximum dose inside the target volume. However, the novel optimized spot placement technique was able to reduce this cost and achieve the same improvements in conformity while maintaining the dose homogeneity of grid-based spot distributions.

The main purpose of this study has been to investigate whether the geometric spot distribution improvements generated by contour-based techniques as shown in **Figure 2** would translate into improved dose distributions. Such improvements were indeed observed to uninvolved tissue outside the target as shown by reduction of integral dose values for both spot sizes and for both targets as shown in **Tables 1** and **2**. The CI was found to be similar between all techniques for a given spot size, except that fewer spots are required to achieve the same level of conformity with the boundary-contoured spot placement techniques. When using a smaller spot size (as is common in the latest generation of proton delivery equipment), minimal changes to the dose distributions were observed and the reported metrics are consistent for all spot placement techniques, except that once again a fewer number of spots are required to achieve these results for the boundary-contoured spot placement techniques. This spot count reduction may be significant enough to have a measurable impact on pencil beam delivery times and/or increase the effectiveness of various types of collimation (static or dynamic) for some delivery systems [11, 12]; as such this work remains relevant for both older- and newer-generation proton delivery systems.

To further explore the dosimetric effects of spot distributions, a simple conical target volume was used. Row 3 of **Figure 1** shows why this conical target provided useful insight into distinguishing the dosimetric impact of spot placement techniques, as a subtle stair-stepped dose pattern along the sides of the cone can be discerned for the first 4 spot placement techniques (when using the large spot size). In contrast, the dose falloff region is smooth and linear, with no obvious “steps” for the optimized spot placement technique. This stepping behavior can be explained by the discrete “jumps” in the grid rows/columns or contour “rings” as the target shape changes with energy layer. For grid-based spot placement techniques, because there is an increase in tumor size from one energy layer to the next, at some point there is a need to add a whole row/column of spots to provide full tumor coverage on that layer. These “row additions” cause a decrease in conformity and lead directly to the stair-stepped dose patterns seen in **Figure 1**. The conical target volume brings this issue to light quite well as the shape (boundary) changes slowly with each energy layer. It is noteworthy that even with the concentric-contours and hybrid techniques, there are still visible steps in the dose along the boundary of the cone in the dose falloff region. These steps are best explained by the same issue of having discrete “row additions” (or an additional band in the concentric-contour scheme) as the target shape changes with each energy layer. And while these additions do not occur on the boundary, they are still near enough to the tumor edges that they remain impactful on the lateral dose falloff. The new optimized spot distributions do not seem to suffer from this issue, as the technique is iterative and when the discrete jumps do occur, they are smoothly redistributed evenly throughout the entire target area, thereby providing a consistent conformity for all energy layers. The conical target volume is also an interesting example to highlight the importance of spot placement on small target areas (those that are approximately 1 spot diameter). In this case, for example, the grid-based schemes required 2 to 3 spots in the deepest layer near the tip of the cone, despite the shape being small enough to use a single spot if properly placed. This is because the grids are not forced to align with the center of the shape for each layer. We implemented the boundary-contoured schemes as they were previously described in the literature [2, 6, 8, 13] and they too required more than a single spot at the cone tip, as the first point was placed on the boundary rather than being centrally located. The new optimized approach, however, contained fallback logic, whereby a single spot would be placed centrally in cases where the target boundary perimeter was smaller than the spot circumference for a given energy layer, creating a more ideal spot placement for small target areas.

In conclusion, this work was performed by using uniform, homogeneous water phantoms with simple target geometries (spherical and conical), and yet noticeable dosimetric benefits were realized through improved proton spot distributions. In real patients, the path of the proton beam will encounter density heterogeneities including air cavities and bones, thereby making even simple patient target volumes quite complex and irregular when viewed on an energy layer-by-layer basis. As these complexities will increase the number of layers required to treat the target volume, each layer's portion of the target volume will be smaller and have a more complex boundary shape. As such, the inclusion of heterogeneities and complex target shapes are expected to increase the benefits seen in this work, as the size of the spots relative to each layer's target volume is increased by such inclusions. However, the interaction with uncertainties involved in patient setup, proton range, and internal motion are not yet clear. Therefore, the next logical expansion of this work would be to perform an analysis similar to that presented here but using real patient computed tomography (CT) data sets and including robustness analysis. Additionally, we recommend further efforts be put forth to expand commercial treatment planning systems to include more advanced spot placement options for routine clinical use.
